# Collagen type 1A1, type 3A1, and LOXL1/4 polymorphisms as risk factors of pelvic organ prolapse

**DOI:** 10.1186/s13104-020-05430-6

**Published:** 2021-01-07

**Authors:** Asuka Ashikari, Tetsuji Suda, Minoru Miyazato

**Affiliations:** 1 Department of Urology, Graduate School of Medicine, University of the Ryukyus, Okinawa, Japan; 2 Department of Systems Physiology, Graduate School of Medicine, University of the Ryukyus, 207 Uehara Nishihara, Okinawa, Japan

**Keywords:** Collagen, Elastin, Single nucleotide polymorphism

## Abstract

**Objective:**

Collagen and elastin are the main components of the female pelvic tissue. We investigated whether single nucleotide polymorphisms (SNPs) of collagen type 1 alpha 1 (COL1A1), collagen type 3 alpha 1 (COL3A1), and lysyl oxidase-like (LOXL) 1 and 4 were associated with the onset of pelvic organ prolapse (POP) in Japanese women. Fifty-two women with POP and 28 women without POP were included. SNPs were identified using the TaqMan® SNP genotyping assay.

**Results:**

Age, parity, and lower urinary tract symptoms were significantly higher in the POP group than in the control group. The prevalence of genotypes with rs2862296 polymorphism of LOXL4, an enzyme essential for extracellular matrix remodeling, was different between the POP (26.9% for GG, 51.9% for AG) and control groups (14.8% for GG, 33.3% for AG). However, polymorphisms of COL1A1, COL3A1, and LOXL1 were not related to the onset of POP. In the multivariate logistic regression analysis, age was significantly associated with the occurrence of POP. In the univariate analysis, LOXL4 polymorphism was associated with the onset of POP in Japanese women. The knowledge of acquired risk factors and polymorphisms in the genomic background of patients with POP may help prevent POP via early conservative interventions.

## Introduction

Pelvic organ prolapse (POP)—herniation of the pelvic organs—is a common disease in the elderly and middle-aged women. They can experience discomfort, pain, voiding disorder, and walking difficulty. Moreover, the quality of life of these women is considerably decreased. POP is caused mainly by acquired risk factors, such as age, obesity, and parity [1, 2]. However, there have been reports of POP development due to genetic factors [3–5] and in women with no acquired risk factors for the condition (i.e., nulliparous or non-obese). It has also been reported that patients with congenital connective tissue disorders, such as Marfan and Ehlers–Danlos syndromes, have a high rate of POP development [6]. Thus, European and American researchers have focused on the development of POP primarily in populations with specific genetic risk factors [7–9]. From Asia, there have been also reported in the genomic risk factor for POP [10–13]. However, research on the relationship between POP and genetic factors in Japanese women is limited. Polymorphisms are influenced by racial genetic backgrounds, therefore, understanding each genetic risk factor associated with POP could help identify patients with a high risk for POP development, and this might help prevent POP by early interventions such as pelvic floor muscle training and weight control.

Anatomically, the pelvic floor consists of fascia, ligaments, muscles, and organs, which are rich in collagen and elastin, with upregulated estrogen expression. Collagen type 1 strengthens the connective tissue, and collagen type 3 and elastin contribute to flexibility. It has been reported that polymorphisms of collagen types 1 and 3 are risk factors for POP with an odds ratio of 1.3 and 4.79, respectively [8, 9]. Lysyl oxidase (LOX) and LOX-like (LOXL) isoenzymes, divided into types 1 to 4, also plays a critical role in the formation and repair of the extracellular matrix, which is responsible for crosslinking between collagen and elastin fibrils [14, 15]. Recently, it has been reported that these enzymes could critically influence cardiovascular function[15]. Although polymorphisms of LOXL1 in humans have not been associated with POP (OR: 1.147) [14], mice lacking LOXL1 do not deposit normal elastic fibers and thus develop POP or emphysema [6]. The functions of LOXL2-LOXL4 related to POP are not well understood. Therefore, in this study, we aimed to study whether polymorphisms of collagen type 1, collagen type 3, LOXL1, and LOXL4 are associated with POP development in Japanese women.

## Main text

### Materials and methods

#### Study population

We recruited subjects from the urology department of a single institution from 2016 to 2019 after obtaining institutional review board approval (approval #226). Women older than 40 years were invited to participate in the study, and an informed consent form was subsequently signed by the participants. The exclusion criteria is women with non-cured cancer. Each subject participated in an interview and underwent clinical examination and Pelvic Organ Prolapse Quantification (POP-Q) system assessment (stages 0 to 4). Eighty women were divided into two groups: 52 women with POP-Q stage 3 or 4 (POP group) and 28 women without POP (POP-Q stage 0 or 1: control group), regardless of whether they were parous or nulliparous. Demographic and clinical data were obtained, including age, body mass index (BMI), parity, family history of POP, and Overactive Bladder Symptom Score (OABSS), which is used to evaluate lower urinary tract syndrome, especially urinary urgency (Table 1).

**Table 1:** Clinical characteristics of patients with POP and those in the control group

		POP group	Control group	*P* value
		n = 52	n = 28	
Age (years)		69.9 ± 7.2	62.8 ± 11.0	0.0035
BMI (kg/m^2^)		24.6 ± 3.2	24.5 ± 4.6	0.4403
Parity (times)		3.3 ± 0.9	2.6 ± 1.5	0.0022
(parity range)		(1–5)	(0–6)	
OABSS		6.0 ± 3.7	3.5 ± 3.9	0.0013
POP-Q stage	≤ I	0	28	
	II	0	0	
	III	31	0	
	IV	21	0	

*BMI* body mass index, *OABSS* overactive bladder symptom score, *POP-Q* pelvic organ prolapse quantification

#### DNA extraction

Whole blood samples of 5 ml were obtained from the 52 women with POP and 28 women without POP. These samples were aliquoted and frozen at – 80 °C until use. Genomic DNA was extracted from the whole blood samples using the ISOSPIN Blood & Plasma DNA (Nippon Gene Co., Ltd., Tokyo, Japan) according to the manufacturer’s instructions.

#### SNP genotyping assays

Single nucleotide polymorphism (SNP) genotyping of COL1A1 (*rs1800012*), COL3A1 (*rs1800255*), LOXL1 (*rs2165241*), and LOXL4 (*rs2862296*) was performed using the TaqMan® SNP Genotyping Assays (Thermo Fisher Scientific, Waltham, MA). A set of predesigned TaqMan SNP Genotyping Assays was used for the analysis of SNPs. The assay ID’s were C_7477170_30, C_7477926_10, C_43559411_10, and sC_1974589_10 (Thermo Fisher Scientific), respectively. Each reaction mixture of 20 µl contained 5 ng (2.5 µl) of genomic DNA, TaqMan Universal PCR Master Mix II (Thermo Fisher Scientific), and TaqMan SNP Genotyping Assay reagents. Real-time polymerase chain reaction (PCR) and the subsequent analysis were performed using the StepOne Plus™ Real-time PCR System (Thermo Fisher Scientific) according to the manufacturer’s instructions. The PCR was performed under the following conditions: 95 °C for 10 min followed by 40 cycles of amplification (denaturation at 92 °C for 15 s, annealing for 60 °C, annealing/extension at 60 °C for 1 min). After amplification, an allelic discrimination analysis was performed using StepOne Software (Thermo Fisher Scientific).

### Statistical analysis

Data are expressed as mean ± standard deviation and analyzed using JMP statistical software (Version 11, SAS Institute, Tokyo, Japan). Comparisons between groups (in terms of age, BMI, parity, and lower urinary tract symptoms) were made using the Mann–Whitney *U* test. Association between polymorphism genotype and POP in the univariate analysis was determined as the odds ratio (OR) with 95% confidence intervals (CI) using a logistic regression model. The cut off of age is 67, that is mean value of total participants. The independent variables in the multivariate analysis were age, parity, BMI, and SNP genotyping for COL3A1 (*rs1800255*), which have been reported to be clinically important factors [9], and LOXL4 (*rs2862296*), which was a significant factor in the univariate analysis in the present study. Results with a *P* value of < 0.05 were considered statistically significant.

## Results

The average age of patients in the POP group was significantly higher than that of patients in the control group (*P* = 0.0035). The parity of the POP group was significantly higher than that of the control group *P* = 0.0022). The lower urinary tract symptoms (OABSS) were significantly higher in the POP group than in the control group (*P* = 0.0013). The prevalence of the CC genotype in the *rs1800012* polymorphism of COL1A1 was 100% in both POP and control groups. For the *rs1800255* polymorphism of COL3A1 in the POP group, the genotypic prevalence was 11.5% for AA, 48.1% for AG, and 40.4% for GG (Table 2). The genotypic frequency of COL3A1 did not differ between the POP and control groups. The prevalence of the *rs2165241* polymorphism of LOXL1 in the POP group was 80.8% for CC, 15.4% for CT, and 3.9% for TT. The genotypic frequency of LOXL1 did not differ between the POP and control groups. On the contrary, for the *rs2862296* polymorphism of LOXL4 in the POP group, the prevalence was 26.9% for GG, 51.9% for AG, and 21.2% for AA. The genotypic frequency of *rs2862296* (AA, AG, and GG) of LOXL4 was significantly different between the POP and control groups (P = 0.0204). In the univariate logistic regression analysis, genotypes GG and AG of LOXL4 (*rs2862296*) were found to be significantly associated with POP (for GG: OR = 4.5, 95% CI: 1.2–19.3, *P* = 0.0239; for AG: OR = 3.8, 95% CI: 1.3–11.8, *P* = 0.0139). There was no relationship between the COL1A1, COL3A1, and LOXL1 SNPs and the presence of POP or severity of the POP-Q stage. In the multivariate logistic regression analysis, age was an independent risk factor associated with the occurrence of POP (OR = 4.56, 95% CI: 1.56–15.6, *P* = 0.006); however, LOXL4 was not an independent risk factor (*P* = 0.172) (Table 3).

**Table 2:** Univariate analysis for association between polymorphisms with and without pelvic organ prolapse

Gene	SNP ID	Allele	POP n (%)	Control n (%)	OR	95% CI	*P* value
COL3A1	*rs1800255*	GG	21 (40.4)	7 (25.9)	1.0 (ref)		
		AG	25 (48.1)	15 (55.6)	0.56	0.18–1.58	0.27
		AA	6 (11.5)	5 (18.5)	0.4	0.1–1.76	0.22
COL1A1	*rs1800012*	CC	52 (100)	28 (100)	–		
		CA	0	0	–		
		AA	0	0	–		
LOXL1	*rs2165241*	CC	42 (80.8)	19 (67.9)	1.0 (ref)		
		CT	8 (15.4)	8 (28.6)	0.45	0.15–1.40	0.17
		TT	2 (3.9)	1 (3.6)	0.9	0.08–20.20	0.94
LOXL4	*rs2862296*	AA	11 (21.2)	14 (51.9)	1.0 (ref)		
		AG	27 (51.9)	9 (33.3)	3.8	1.30–11.80	0.01
		GG	14 (26.9)	4 (14.8)	4.5	1.20–19.30	0.02

*SNP* single nucleotide polymorphisms, *ref* reference

**Table 3:** Multivariate logistic regression analysis for association between clinical backgrounds and polymorphism with and without pelvic organ prolapse

Variables		OR	95% CI	P-value
Age (years)	67 >			
	67 ≤	4.56	1.56–15.6	0.0060
BMI (kg/m^2^)	25 >			
	25 ≤	1.91	0.61–6.37	0.2651
Parity (times)	3 >			
	3 ≤	1.67	0.48–5.65	0.4135
Gene	SNP ID			P-value
COL3A1	*rs1800255*			0.4128
LOXL4	*rs2862296*			0.1712

*SNP* single nucleotide polymorphisms, *BMI* body mass index

## Discussion

An understanding of genetic risk factors, in addition to acquired risk factors, can help improve the treatment of POP. Therefore, in this study, we investigated the genetic polymorphisms associated with POP in Japanese women.

Age and parity were significantly higher in the POP group than in the control group, as previously reported [1, 2]. The patients with POP also presented more lower urinary tract symptoms. In the univariate analysis, the polymorphism of LOXL4, an enzyme that acts in collagen and elastin fiber maturation and is essential for extracellular matrix remodeling, was found to be associated with the onset of POP in Japanese women. However, the polymorphisms of COL1A1, COL3A1, and LOXL1 were not related to the onset of POP. To the best of our knowledge, this is the first study to report that LOXL4 polymorphism might be associated with POP development in Japanese women; this has not been reported in other ethnic populations.

In mammalian genomes, five LOX enzymes have been identified (LOX and LOXL1 to LOXL4) [16]. LOX and LOXLs control cell proliferation, differentiation, and transcriptional regulation, thereby, alteration has been reported to be related to the onset of cardiovascular diseases [15]. Liu et al. also reported that mice lacking the LOXL1 did not deposit normal elastic fibers in the uterine tract post-partum and developed POP, enlarged air spaces in the lung, loose skin, and vascular abnormalities with concomitant tropoelastin accumulation [6]. However, the precise roles of LOXL2 to LOXL4 on elastin and collagen sub-states are not well known. In the present study, the genotype frequency of *rs2862296* (AG and GG) of LOXL4 was significantly higher in the POP group than in the control group. It has been reported that LOXL4, encoded by a gene located at 10q24-26 [17], has a direct role in transforming growth factor β1 signaling and contributes to the vascular process associated with extracellular matrix remodeling and fibrosis [18]. Therefore, LOXL4, as well as LOXL1, may serve as a cross-linking enzyme and an element of the scaffold to ensure elastin deposition. Thus, the polymorphism of LOXL4 might be associated with the onset of POP in Japanese women.

Collagen types 1 and 3 are the two main components of pelvic connective tissue. Type 1 fibers are well-organized and are present in the uterosacral ligaments that provide DeLancy level I support to the cervix and vaginal apex [8, 19]. Type 3 fibers are more prominent in the loose areolar tissue surrounding the vagina and pelvic organs. In a systematic review of the genetic epidemiology of POP, Ward et al. suggested that COL3A1 (*rs1800255*) genotype AA is associated with POP (OR = 4.79, 95% CI 1.91–11.98, *P* = 0.001) [8]. Furthermore, another systematic review reported that COL1A1 (*rs1800012*) polymorphism may be associated with prolapse (OR = 1.3, 95% CI 1.0–1.7, *P* = 0.133) [9]. However, our study did not reveal the relationship between the genes encoding collagen types 1–3 and POP. The prevalence of the CC genotype in the *rs1800012* polymorphism of COL1A1 was 100% in both POP and control groups. These results are consistent with those reported in the genome cohort study by the Tohoku Medicine Megabank Organization. Thus, the genetic differences in different populations worldwide may have affected the COL3A1 (*rs1800255*) and COL1A1 (*rs1800012*) polymorphisms; further studies are needed to clarify the association of genomic polymorphisms with POP.

In conclusion, in the present study, we could find, LOXL4 *rs2862296* polymorphism was associated with POP in Japanese women in the univariate analysis (G allele 53%). The knowledge of acquired risk factors and racial genomic background of patients with POP can help improve surgical indications or to prevent POP via early conservative intervention. We are planning a large-scale clinical study to examine the genome-wide association studies (GWAS) of Japanese patients with POP.

## Limitations

The main limitations of this study were the small sample size and the fact that the samples were all from the Okinawan population, which has several genomic differences compared with the population in mainland Japan, east Asia. Because of small sample size, the CIs have wide range (Tables 2 and 3). Additionally, we chose only collagen and elastin polymorphisms, although other genomic variations such as estrogen receptor α [20] or matrix metalloproteinase [21] genes have been reported. Moreover, *rs2862296* (LOXL4), whose wild-type allele is A in Japanese women, has been reported in Brazilian women [19]. However, there were several variations in the distribution of the reference allele. In the multivariate analysis, LOXL4 was not an independent risk factor, but presented a tendency to be associated with the onset of POP (*P* = 0.172). Despite these limitations, we believe that genetic variations in the *LOXL4* gene may play a role in the susceptibility of women to POP.

## Data Availability

The data sets of the current study are available from the corresponding author on reasonable request.

## Abbreviations

POP: Pelvic organ prolapse
LOX: Lysyl oxidase
LOXL: LOX-like
POP-Q: Pelvic Organ Prolapse Quantification
BMI: Body mass index
OABSS: Overactive Bladder Symptom Score
SNP: Single nucleotide polymorphism
PCR: Real-time polymerase chain reaction
OR: Odds ratio
